# Associations between parents' exposure to a multisectoral programme and infant and young child feeding practices in Nepal

**DOI:** 10.1111/mcn.13143

**Published:** 2021-07-09

**Authors:** Kenda Cunningham, Devin Nagle, Poonam Gupta, Ramesh Prasad Adhikari, Sujata Singh

**Affiliations:** ^1^ Helen Keller International New York New York USA; ^2^ Department of Population Health, Faculty of Epidemiology, London School of Hygiene and Tropical Medicine London England; ^3^ Heilbrunn Department of Population and Family Health, Mailman School of Public Health Columbia University New York New York USA; ^4^ Department of International Health, Johns Hopkins Bloomberg School of Public Health Johns Hopkins University Baltimore Maryland USA; ^5^ Helen Keller International Lalitpur Nepal; ^6^ CARE Lalitpur Nepal

**Keywords:** child nutrition, family approach, infant and young child feeding, minimum acceptable diet, minimum dietary diversity, Nepal, sick child feeding

## Abstract

In Nepal, an at‐scale, multisectoral programme—*Suaahara* (2011–2023)—aims to improve nutrition behaviours. *Suaahara II* (2016–2023) transitioned from a mother/child dyad focus to explicitly targeting all family members. Evidence is scant, however, regarding how exposure by men to social and behaviour change interventions relates to nutrition outcomes. This study uses a 2019 cross‐sectional monitoring dataset to test associations between maternal and male household head exposure to *Suaahara II* interventions (interacting with a frontline worker, participating in a community event or listening to the *Bhanchhin Aama* radio programme) and adoption of three infant and young child feeding practices: minimum dietary diversity, minimum acceptable diet and sick child feeding, in households with a child under 2 years (*n* = 1827). Maternal exposure to *Suaahara II* had a positive association with minimum dietary diversity (OR: 1.71, 95% CI [1.27, 2.28], *P* < 0.001), minimum acceptable diet (OR: 1.60, 95% CI [1.19, 2.14], *P* = 0.002) and increased feeding to a sick child (OR: 2.11, 95% CI [1.41, 3.17], *P* < 0.001). Male household head exposure was only associated with increased feeding to a sick child (OR: 2.21, 95% CI [1.27, 3.84], *P* = 0.005). Among households with an exposed mother, having an exposed male household head nearly tripled the odds of appropriate sick child feeding (OR: 2.90, 95% CI [1.57, 5.34], *P* = 0.001) but was not significantly associated with the other two outcomes. These findings suggest that the relationships between exposure to nutrition programmes and outcomes are complex and further research is needed to understand variation by family member, behavioural outcome and context.

**Key messages**

- Approximately three‐fourths of mothers and more than one‐third of male household heads were exposed to a multisectoral nutrition programme 2 years after the programme started.
- Positive associations between maternal exposure to *Suaahara II* and minimum dietary diversity, minimum acceptable diet and sick child feeding were found.
- Male household head exposure to *Suaahara II* interventions was associated with an increased odds of 2.21 for appropriate sick child feeding and nearly tripled the odds of this ideal practice in households with an exposed mother, but no association was found between male household head exposure and either minimum dietary diversity or minimum acceptable diet.
- These findings suggest that the family approach may be effective at increasing adoption of some ideal complementary feeding practices but that more research is needed to understand how engaging male family members relates to adoption of ideal child nutrition practices in different contexts.

## 1 INTRODUCTION

### 1.1 A family approach for nutrition

It is widely agreed that the thousand‐day period encompassing the 9 months of pregnancy plus the first 2 years of an infant's life is a window of opportunity for interventions, precisely because nutritional well‐being at this critical time will impact the child for the rest of his or her life (Beluska‐Turkan et al., 2019; Moore, Arefadib, Deery, & West, 2017; Schwarzenberg & Georgieff, 2018). Insufficient nutrition during this period can lead to a plethora of health and development risks including stunting, wasting, brain underdevelopment and death (Kabaran, 2018; Moore, Arefadib, Deery, & West, 2017).

Improving maternal and child health and nutrition (MCHN) outcomes in low‐ and middle‐income countries is a high priority of governments and development partners alike (World Health Organization & Maternal Newborn and Child Health Network for Asia and the Pacific, 2009). MCHN programmes and policies have traditionally targeted mothers, but a family approach is increasingly being promoted (Hingle, O'Connor, Dave, & Baranowski, 2010). Rather than focus exclusively on mothers, programmes and policies engage the whole family, specifically adult decision makers, in recognition that mothers do not often make decisions or control financial resources alone and that care for herself and her children is a whole family responsibility (Berger & Font, 2015).

Research on family approaches to MCHN interventions is emerging, dominated by qualitative studies to date. Evidence suggests that breastfeeding interventions that target the father improve breastfeeding initiation, duration and exclusivity rates in a variety of settings (Abbass‐Dick, Brown, Jackson, Rempel, & Dennis, 2019; Mahesh et al., 2018; Martin et al., 2020; Mitchell‐Box & Braun, 2013). Training fathers to be more involved and supportive of continued breastfeeding was found to be associated with higher breastfeeding and exclusive breastfeeding rates (Raeisi, Shariat, Nayeri, Raji, & Dalili, 2014). Fewer studies have been done on complementary feeding outcomes. A cross‐sectional study in Ethiopia found positive associations between fathers' knowledge and attitudes and child dietary diversity (Bilal et al., 2016). A quasi‐experimental study in western Kenya found small but significant, positive associations between improving knowledge of optimal infant feeding practices and encouraging provision of additional social support from the father and grandmother and child dietary diversity, but no associations for child minimum acceptable diet (Mukuria, Martin, Egondi, Bingham, & Thuita, 2016).

This evidence, however, on the effectiveness of engaging fathers, or other male family members, in interventions as a way of improving MCHN is limited, particularly in South Asia. The need for studies that assess interventions targeted only at mothers versus others in the family and community has been documented (Fox, Davis, Downs, Schultink, & Fanzo, 2019). With limited resources for interventions to be implemented at scale, and the additional resources required to incorporate a family approach, these studies of the benefits of different approaches are needed to guide donors, governments and programme implementers.

### 1.2 The Nepali context and *Suaahara II*

Nepal has made significant progress in reducing maternal and child mortality and improving health outcomes, including reducing maternal and child undernutrition (Cunningham, Headey, Singh, Karmacharya, & Rana, 2017). Nevertheless, according to Nepal's 2016 Demographic and Health Survey, among children 6–23 months of age, only 47% are fed a diet of foods from at least four of seven food groups that meets the cut‐off for minimum dietary diversity and only 37% are fed a minimum acceptable diet (Ministry of Health of Nepal et al., 2017). Additionally, among children under the age of 5 with diarrhoea in the 2 weeks before the survey, 27% were given less food during their illness rather than the appropriate practice of feeding a child with diarrhoea the same or more food than usual (Ministry of Health of Nepal et al., 2017). The Government of Nepal implements a Multi‐Sectoral Nutrition Plan (MSNP), now in its second phase, prioritizing the reduction of malnutrition.

*Suaahara II*, a 7‐year (2016–2023) USAID‐funded multisectoral nutrition programme that supports Nepal's MSNP, builds off of the first 5‐year phase of the programme (2011–2016) and operates in 42 districts of Nepal to reach over 1 million households in the 1000‐day period (Helen Keller International, 2018c). *Suaahara II*'s overall aim is to improve the nutritional status of pregnant and lactating women and children under 2 years via interventions spanning maternal, newborn, and child nutrition; health and family planning services; water, sanitation and hygiene (WASH); homestead food production and agricultural marketing; and nutrition governance (U.S. Agency for International Development, 2017). *Suaahara II* also uses cross‐cutting approaches to address gender equality and social inclusion (GESI) and monitoring, evaluation and research (MER) for learning. *Suaahara II* works at national and subnational levels to improve nutrition policies and stakeholder coordination and supports a cadre of Government of Nepal health, agriculture and WASH frontline workers to improve quality and inclusive services. *Suaahara II* household and community programming primarily operates via social and behaviour change communication platforms including interpersonal communication, community mobilization, mass media and more recently SMS messages and social media. *Suaahara II* hired and trained field staff to conduct home visits and facilitate community events such as food and handwashing demonstrations, usually done during health mothers' group meetings, in collaboration with Nepal's Female Community Health Volunteers. The mass media edutainment radio programme, titled *Bhanchhin Aama* (*Mother Knows Best*), covers the wide range of practices that *Suaahara II* seeks to influence. These interventions target mothers and family members in 1000‐day households to reinforce key messages and promote optimal nutrition‐related practices (Pun et al., 2019; Suresh et al., 2019).

In the fall of 2016, *Suaahara II'*s strategy started transitioning from a mother/child dyad focus to a whole family approach, recognizing the potential of engaging the entire family in improving MCHN (Pun et al., 2019). This strategy was informed by learnings from *Suaahara I* implementation and early *Suaahara II* formative research that found that, in most of households, even when mothers had primary responsibility, for a task she asked her family members for input (Helen Keller International, 2018a). Likewise, mothers with young children suggested that *Suaahara II* also send SMS messages to their husbands and mothers‐in‐law since they too are key decision makers regarding maternal and child health and nutrition (Helen Keller International, 2018b). To shift to this family approach, *Suaahara II* trained its frontline workers (FLWs) and developed a toolkit to provide interpersonal communication to all family members instead of only mothers and to encourage the whole family to participate in community events. *Bhanchhin Aama* (*Mother Knows Best*) was also revised in July 2017 to include new characters, namely, an adolescent girl and grandparents, to expand the programme's appeal to the whole family.

To date, however, the benefits and risks of the transition to this family approach vis‐à‐vis MCHN behaviours that the programme aims to improve are unknown. There is scant evidence on family approaches to nutrition programming, especially those engaging multiple adult family members on multiple MCHN outcomes. This manuscript starts to fill these gaps by testing associations between exposure to *Suaahara II* by both mothers and male heads of household and adoption of ideal infant and young child feeding (IYCF) practices in households with a child under 2 years after roll‐out of the family approach. This study further explores the association between male household head exposure and these key outcomes limited to households where the mother is exposed to test the theory that two exposed adults, versus one, improves the odds of adopting the ideal behaviours.

## 2 METHODS

### 2.1 Survey design and sampling

This paper used a cross‐sectional monitoring dataset for an at‐scale, integrated programme in Nepal, known as *Suaahara II*. The general objective of this cross‐sectional monitoring survey is to monitor the progress of key maternal and child health and nutrition indicators over time. The data were collected from June to September in 2019 across all three agro‐ecological zones—mountains, hills and *terai* (plains)—and are representative of the 42 of Nepal's 77 districts in which *Suaahara II* interventions are implemented. Multistage cluster sampling was used to randomly select 16 districts; 32 municipalities (one rural and one urban per district); 96 wards (three per municipality); 192 subwards (two per ward); and 3648 households with a child under 5 years (19 per cluster). Probability proportional to size (PPS) sampling was employed to select study areas, followed by a listing of all households with a child under the age of 5 with the child's mother in residence and 19 of these eligible households per cluster randomly drawn from a bowl. In each selected household, the youngest child under 5 years was selected as the child for the study.

The primary survey respondents were the mother of the selected child and the head of household, male if available. The mothers' questionnaire asked questions on practices related to MCHN topics such as antenatal care, delivery and postnatal care, childcare, and IYCF, as well as general health seeking, agriculture and homestead food production, and WASH. The household heads' questionnaire asked questions on household demographics and economics, household food security, land use and agricultural practices, and included observations on the household structure. Both surveys included a 24‐h dietary recall and asked about various domains of household‐level empowerment, integrated nutrition knowledge and exposure, self‐efficacy, gender‐based violence and psychosocial well‐being, and *Suaahara II* exposure.

### 2.2 Measurements and variables

The primary exposure variable was maternal and male household head exposure to three key types of *Suaahara II* interventions: (1) met with a *Suaahara II* FLW in the past 6 months (yes/no); (2) ever participated in a *Suaahara II* community event such as a food demonstration (yes/no); and (3) ever listened to radio programme *Bhanchhin Aama* (yes/no). Given low levels of exposure to each type of intervention, a final summary binary variable was created to measure exposure to any of these three intervention types (yes/no; Suresh et al., 2019).

The three primary outcome variables are all key IYCF behaviours promoted by *Suaahara II* due to their importance for improved child nutritional well‐being:

1. Minimum dietary diversity: The World Health Organization (WHO) developed an indicator for child minimum dietary diversity to measure the proportion of children between 6 and 23 months of age who consume foods from 4 or more of 7 food groups (grains; pulses; dairy; flesh foods; eggs; vitamin‐A rich fruits and vegetables; and other fruits and vegetables) over a 24‐hour dietary recall period (World Health Organization, 2008a, 2008b). An open‐ended 24‐h dietary recall was asked so that mothers could report what foods the child had consumed in the 24 h prior to the survey (or day before that, if yesterday had been a holiday or otherwise unusual day regarding foods consumed). Foods consumed were aggregated into the seven food groups and each child's dietary diversity score was created as a simple sum of the number of food groups. A binary variable was created to measure whether the child who was 6 to 23 completed months of age had consumed foods from four or more of the food groups and therefore met minimum dietary diversity (yes/no);
2. Minimum acceptable diet: WHO developed an indicator for child minimum acceptable diet to measure the proportion of children 6 to 23 completed months of age who receive a minimum acceptable diet (apart from breast milk), meaning that the child had at least the minimum dietary diversity and the minimum meal frequency during the previous day (World Health Organization, 2008a, 2008b); and
3. Sick child feeding: UNICEF recommends that sick children are fed more than usual (UNICEF, 2020). A binary variable was created for those who had been sick in the past 2 weeks to denote if the mother reported to have fed more during illness (yes/no).

Various socio‐economic and demographic factors which were potential confounders of the associations of interest were identified based on a literature review and knowledge of the local context and included in analyses. These included the following household level factors: whether more than one child under 5 years lived in the house (yes/no); the agro‐ecological zone of residency (mountains, hills and *terai*); whether the household was a member of a socially excluded caste or ethnic group defined as Dalit, Muslim or disadvantaged Janajati (yes/no); whether the household reported food insecurity (yes/no); and the socioeconomic status of the household (measured in quintiles, using the EquityTool, a tool that uses a short survey to measure relative wealth among a survey population in relation to the national averages. For Nepal, the Demographic and Health Survey data from 2016 is used and a household score is calculated based on ownership of a fan, chair, table, sofa, cupboard and television along with materials that make up the roof, walls and floors, and major cooking fuel source (Metrics for Management, 2018). Other demographic factors of interest were maternal and male household head age (in completed years) and years of schooling (in completed years). Child's sex (male/female) and age (in completed months) were also included.

### 2.3 Data analysis

As this study focused on behaviours that are important for child nutritional well‐being during the 1000‐day period, we restricted our analysis to households with a child 0–23.9 months of age (*n* = 1827). Only about half of these households had a male household head (n = 942) respond to the survey. Therefore, the sample sizes vary for each model, depending on the exposure variable (which household member) as well as the outcome variable. We conducted reverse power calculations to estimate the minimum detectable effect size for each of these models (Table S1A).

Descriptive analyses were performed to produce percent distributions, means and standard deviations for socio‐economic and demographic variables, primary outcome variables, and primary exposure variables. These analyses were conducted among all households with a child under 24 months of age, along with separate models run for households with and without a male household head. Differences between households with and without a male household head were tested using chi‐squared tests of independence for binary/categorical variables and independent *t* tests for continuous variables.

Logistic regression models were first run to estimate associations between exposure by mothers and male household heads and outcomes of interest separately. We then used logistic regression models, limited to households where the mother was exposed to at least one of the three *Suaahara II* interventions (*n* = 1364), to estimate associations between *Suaahara II* exposure by the male household head and all outcomes.

All analyses were adjusted for the individual and household level potential confounding factors mentioned above. All models were also adjusted for clustering. All statistical analyses were performed using Stata version 16.1 (2020).

### 2.4 Ethical approval

Ethics approval was obtained from the Nepal Health Research Council (NHRC) in 2019. Respondents gave written informed consent to participate in the survey and reaffirmed their consent to continue their survey after completing each module in the questionnaires before continuing to the next module.

## 3 RESULTS

### 3.1 Household characteristics and key outcomes of sample

Household characteristics and key outcomes among sample households overall, as well as disaggregated between those with and without a male household head, are presented in Table 1. Nearly three‐fourths of households had only one child under 5 years (73%). Slightly over one‐third (37%) of households were from upper caste groups (Brahmin/Chhetri); less than half of households (39%) were in the bottom two equity quintiles. Almost two‐thirds of households (62%) had extended family members residing in their household. Mothers had, on average, 7.4 years of schooling, whereas male household heads had, on average, 6.8 years of schooling. Nearly three‐fourths of male household heads (74%) were also the father of the young child.

**TABLE 1:** Household characteristics and infant and young child feeding practices

	All	Households with male household head	Households without male household head	Statistical testing of differences
	*N* = 1827	*N* = 942	*N* = 885	
	% or mean (SD)	% or mean (SD)	% or mean (SD)	*P* value
**Household characteristics**				
Only 1 child <5 years of age	73.3%	74.1%	72.5%	0.452
Agro‐ecological zone of residency				0.102
Hills	55.7%	53.3%	58.2%	
Mountain	13.0%	13.5%	12.5%	
*Terai*	31.3%	33.2%	29.3%	
Disadvantaged/remote community	27.9%	26.2%	29.7%	0.096
Caste/ethnicity: Brahmin/Chhetri	36.8%	33.1%	40.8%	0.001
Household owns a radio	28.4%	33.7%	22.7%	<0.001
Equity quintile				<0.001
Lowest	15.7%	13.2%	18.4%	
Second lowest	23.3%	20.2%	26.6%	
Middle	22.2%	24.5%	19.7%	
2nd highest/highest	38.8%	42.1%	35.4%	
Household structure (*N* = 1821, 939, 882)				<0.001
Mother only	16.0%	—	33.0%	
Mother and husband only	21.6%	26.8%	16.0%	
Extended family	62.4%	73.2%	51.0%	
**Child characteristics**				
Sex: Boy	53.9%	54.1	53.6	0.803
Age (months; range 0–23.9)	11.7 (7.0)	11.0 (7.1)	12.3 (6.8)	<0.001
Age group				0.001
0–5.9 months	23.6%	27.1%	19.9%	
6–11.9 months	26.4%	27.0%	25.8%	
12–17.9 months	24.3%	23.0%	25.7%	
18–23.9 months	25.7%	22.9%	28.7%	
Wellness: no sickness in last 2 weeks	59.3%	60.4%	58.1%	0.312
**Maternal characteristics**				
Age (years: range 15–49)	24.6 (4.8)	24.6 (4.8)	24.6 (4.8)	0.994
Years of schooling completed	7.4 (3.8)	7.6 (3.7)	7.1 (3.8)	0.001
**Male household head characteristics**				
Age (years: range 18–92)	35.6 (13.9)	35.6 (13.9)	—	—
Years of schooling completed	6.8 (4.0)	6.8 (4.0)	—	—
Father is male household head	73.7%	73.7%	—	—
**Child health and nutrition practices**				
Minimum dietary diversity (*N* = 1396, 687, 709)	57.5%	57.5%	57.4%	0.972
Minimum acceptable diet (*N* = 1396, 687, 709)	47.2%	46.4%	48.0%	0.569
Sick child feeding: more during illness (*N* = 597, 283, 314)	35.9%	35.0%	36.6%	0.676

Households with a male household head in residence, compared to households without a male household head, were found to have a higher prevalence of radio ownership (34% vs. 23%, *P* < 0.001), were from higher equity quintiles on average (67% vs. 55% in the upper two equity quintiles, *P* < 0.001), and more resided in extended families (73% vs. 51%, *P* < 0.001). Mothers in the households with a male household member in residence were slightly more educated (7.6 vs. 7.1 years, *P* < 0.001). Children were slightly younger in households with a male household head (11 vs. 12 months), but neither child wellness nor IYCF outcomes differed overall in these two types of households (*P* < 0.001).

### 3.2 Exposure to *Suaahara II*

Exposure to *Suaahara II* interventions by both mothers and male household heads is presented in Table 2. In 2019, after 2 years of *Suaahara II* implementation, nearly three‐fourths of mothers with a child under 2 years had been reached by at least one of the three types of programme interventions. Interaction with a frontline worker in the 6 months prior to the survey was 41%, participation in a *Suaahara II*‐facilitated community event was 48% and ever listened to *Bhanchhin Aama* was 51%.

**TABLE 2:** Maternal and male household head exposure to *Suaahara II*

	Maternal	Male household head	Statistical testing of differences
	2019	2019	
	*N* = 1826	*N* = 941	
	%	%	*P* value
Any *Suaahara* intervention	74.7%	36.5%	<0.001
**Specific *Suaahara* interventions**			
Interaction with *Suaahara* FLW in past 6 months	41.1%	9.0%	<0.001
Participated in any community event (food demo, etc.)	48.0%	3.5%	0.003
Listened to *Bhanchhin Aama*	51.3%	31.0%	<0.001
**Degree of exposure to each platform**			
**Number of FLW interactions (in last 6 months)**			<0.001
0	58.9%	91.0%	
1	20.1%	5.5%	
2 or more	21.0%	3.5%	
**Number of community events (ever)**			0.033
0	52.0%	96.5%	
1	32.3%	3.0%	
2 or more	15.7%	0.5%	
**Frequency of listening to *Bhanchhin Aama***			<0.001
Never	48.7%	69.0%	
Less than once a month	30.3%	19.0%	
Once a month or more	21.0%	12.0%	
**Scale of exposure**			<0.001
0	25.3%	63.6%	
1	28.6%	30.1%	
2 or more	46.1%	6.4%	

On the other hand, over one‐third of male household heads (37%) had been exposed to at least one of the three types of *Suaahara II* interventions. Less than one in 10 male household heads (9%) reported an interaction with a frontline worker in the past 6 months and only 4% had ever participated in a community event. The bulk of exposure was from ever having listened to *Bhanchhin Aama*, with nearly one‐third (31%) of male household heads reporting having listened.

### 3.3 Associations between exposure to *Suaahara II* and child nutrition outcomes

Table 3 presents results from logistic regression analyses examining associations between mothers and male household heads to *Suaahara II* and three key IYCF outcomes.

**TABLE 3:** Associations between maternal and male household head exposure to *Suaahara II* and infant and young child feeding practices

	Minimum dietary diversity (6–23.9 months)			Minimum acceptable diet (6–23.9 months)			Sick child feeding (0–23.9 months)		
	OR	95% CI	*P* value	OR	95% CI	*P* value	OR	95% CI	*P* value
**Mother**	(*N* = 1396)			(*N* = 1396)			(*N* = 744)		
Any *Suaahara* exposure	1.71	1.27–2.28	<0.001	1.60	1.19–2.14	0.002	2.11	1.41–3.17	<0.001
Maternal age (range: 15–49)	1.00	0.97–1.02	0.732	0.98	0.96–1.01	0.123	0.98	0.95–1.02	0.310
Maternal education (years, range: 0–13)	1.00	0.99–1.00	0.037	1.00	0.99–1.00	0.097	1.00	0.99–1.01	0.952
Child sex (male)	1.10	0.89–1.36	0.367	1.08	0.88–1.31	0.470	0.95	0.72–1.26	0.739
Child age group (ref: 0–5.9 months)									
6–11.9 months							1.27	0.81–1.98	0.299
12–17.9 months	1.90	1.49–2.44	<0.001	1.89	1.45–2.45	<0.001	1.54	0.94–2.51	0.084
18–23.9 months	2.66	2.02–3.50	<0.001	1.62	1.20–2.17	0.001	1.02	0.61–1.71	0.925
Wellness: no sickness in last 2 weeks	1.39	1.11–1.73	0.003	1.22	0.98–1.53	0.075			
Only 1 child <5 years of age	1.09	0.83–1.44	0.538	0.99	0.75–1.30	0.934	0.79	0.54–1.16	0.226
Household structure: mom and other adult present	1.06	0.79–1.42	0.700	0.97	0.74–1.28	0.832	1.21	0.79–1.85	0.388
Household agroecological zone (ref: Hills)									
Mountains	1.56	1.05–2.31	0.028	1.78	1.21–2.62	0.004	0.90	0.49–1.64	0.729
*Terai*	0.59	0.43–0.82	0.002	0.76	0.55–1.05	0.098	0.83	0.53–1.29	0.404
Disadvantaged/remote community	0.95	0.70–1.30	0.760	1.07	0.82–1.41	0.610	0.70	0.45–1.09	0.114
Caste/ethnicity: Brahmin/Chhetri	1.33	1.03–1.71	0.028	1.15	0.90–1.48	0.260	1.00	0.67–1.48	0.992
Household owns a radio	1.22	0.93–1.59	0.148	1.24	0.97–1.59	0.092	1.29	0.91–1.82	0.150
Household equity quintile (ref: lowest)									
Second lowest	1.33	0.90–1.94	0.149	1.21	0.82–1.77	0.335	0.90	0.56–1.43	0.645
Middle	1.57	1.06–2.32	0.024	1.24	0.84–1.82	0.281	1.17	0.69–1.99	0.557
Second highest/highest	1.77	1.23–2.56	0.002	1.27	0.89–1.83	0.186	1.19	0.72–1.95	0.500
**Male head of household**	**(*N* = 687)**			**(*N* = 687)**			**(*N* = 372)**		
Any *Suaahara* exposure	1.18	0.80–1.74	0.399	1.03	0.72–1.45	0.881	2.21	1.27–3.84	0.005
Male household head age (range: 18–84)	1.01	0.98–1.03	0.512	1.01	0.98–1.03	0.671	1.00	0.96–1.05	0.946
Male household head education (range: 0–13)	1.04	0.99–1.10	0.140	1.04	0.99–1.09	0.169	0.94	0.87–1.02	0.133
Maternal age (range: 15–49)	0.99	0.96–1.03	0.777	0.98	0.95–1.02	0.396	0.95	0.89–1.01	0.109
Maternal education (range: 0–13)	0.99	0.99–1.00	0.050	0.99	0.98–1.00	0.040	0.99	0.98–1.00	0.154
Father is male household head	0.99	0.49–1.99	0.974	0.93	0.47–1.84	0.836	1.45	0.37–5.72	0.596
Child age group (ref: 0–5.9 months)									
6–11.9 months							0.99	0.54–1.80	0.975
12–17.9 months	2.09	1.38–3.17	0.001	2.19	1.46–3.28	<0.001	1.54	0.85–2.77	0.153
18–23.9 months	2.56	1.70–3.86	<0.001	1.36	0.90–2.03	0.141	0.67	0.33–1.37	0.277
Child sex (male)	1.08	0.80–1.46	0.617	1.03	0.76–1.41	0.843	1.27	0.79–2.04	0.318
Wellness: no sickness in last 2 weeks	1.65	1.19–2.28	0.003	1.43	1.04–1.95	0.025			
Only 1 child <5 years of age	1.43	0.98–2.08	0.062	1.18	0.81–1.71	0.384	0.81	0.45–1.48	0.499
Household agroecological zone (ref: Hills)									
Mountains	1.26	0.80–2.01	0.322	1.61	1.03–2.52	0.035	1.27	0.56–2.87	0.572
Terai	0.40	0.25–0.62	<0.001	0.58	0.37–0.91	0.019	0.62	0.33–1.15	0.130
Disadvantaged/remote community	0.76	0.50–1.15	0.193	0.96	0.63–1.47	0.855	0.51	0.26–1.00	0.050
Caste/ethnicity: Brahmin/Chhetri	1.26	0.85–1.87	0.241	1.13	0.76–1.66	0.550	1.12	0.65–1.93	0.684
Household owns a radio	1.26	0.87–1.82	0.224	1.29	0.90–1.83	0.162	1.11	0.70–1.76	0.663
Household equity quintile (ref: lowest)									
Second lowest	1.43	0.80–2.57	0.228	1.32	0.76–2.29	0.330	1.15	0.46–2.88	0.767
Middle	1.74	1.02–2.97	0.043	1.46	0.86–2.49	0.159	1.01	0.41–2.47	0.979
Second highest/highest	1.76	1.04–2.96	0.035	1.41	0.85–2.34	0.179	1.62	0.66–4.00	0.292

In the adjusted regression models, we found maternal exposure to *Suaahara II* was positively associated with all three of the child nutrition outcomes: minimum dietary diversity (OR: 1.71, 95% CI [1.27, 2.28], *P* < 0.001), minimum acceptable diet (OR: 1.60, 95% CI [1.19, 2.14], *P* = 0.002), and increased feeding to the child when s/he is sick (OR: 2.11, 95% CI [1.41, 3.17], *P* < 0.001). Male household head exposure to *Suaahara II*, however, was only associated with one of the three child nutrition outcomes: increased feeding to the child when s/he is sick (OR: 2.20, 95% CI [1.27, 3.84], *P* = 0.005).

Table 4 presents results from the logistic regression analyses examining associations between male household head exposure to *Suaahara II* and the three IYCF outcomes, only in households where the mother was exposed to enable testing to compare whether exposure by more than one adult family member (versus one) increases the odds of adoption of the promoted practices. Among households where the mother was exposed to *Suaahara II*, in final adjusted models, we found a positive, significant association between male household head exposure and a nearly three‐fold increase in feeding the child more food when s/he is sick (OR: 2.90, 95% CI [1.57, 5.34], *P* = 0.001). We found no significant associations between male household exposure, in households where mothers have been exposed, and the other two IYCF outcomes.

**TABLE 4:** Associations between male household head exposure to *Suaahara II* and infant and young child feeding practices, in households with exposed mothers

	Minimum dietary diversity (6–23.9 months)			Minimum acceptable diet (6–23.9 months)			Sick child feeding (0–23.9 months)		
	OR	95% CI	*P* value	OR	95% CI	*P* value	OR	95% CI	*P* value
**Male head of household**	(*N* = 496)			(*N* = 496)			(*N* = 259)		
Any *Suaahara* exposure	1.21	0.76–1.92	0.419	1.02	0.69–1.52	0.917	2.90	1.57–5.34	0.001
Male household head age (range: 18–84)	1.00	0.97–1.04	0.848	1.00	0.97–1.03	0.843	1.04	0.97–1.10	0.244
Male household head education (range: 0–13)	1.06	0.99–1.14	0.090	1.04	0.98–1.10	0.236	0.93	0.84–1.03	0.144
Maternal age (range: 15–49)	1.00	0.95–1.05	0.909	0.99	0.94–1.03	0.587	0.92	0.84–1.00	0.051
Maternal education (range: 0–13)	0.99	0.97–1.00	0.038	0.99	0.97–1.00	0.081	0.99	0.98–1.00	0.169
Father is male household head	0.75	0.30–1.85	0.530	0.78	0.32–1.88	0.573	3.89	0.61–24.75	0.151
Child age group (ref: 0–5.9 months)									
6–11.9 months	‐	‐	‐	‐	‐	‐	0.67	0.31–1.44	0.306
12–17.9 months	2.49	1.51–4.10	<0.001	2.63	1.65–4.18	<0.001	1.23	0.60–2.55	0.573
18–23.9 months	2.26	1.36–3.74	0.002	1.11	0.69–1.79	0.656	0.42	0.18–0.95	0.038
Child sex (male)	1.11	0.77–1.60	0.585	1.06	0.73–1.53	0.755	1.55	0.86–2.81	0.148
Wellness: No sickness in last 2 weeks	1.53	1.03–2.26	0.034	1.40	0.95–2.05	0.089	‐	‐	‐
Only 1 child <5 years of age	1.46	0.93–2.30	0.098	1.16	0.75–1.79	0.518	1.09	0.53–2.23	0.818
Household agroecological zone (ref: Hills)									
Mountains	1.41	0.80–2.48	0.239	1.75	1.00–3.04	0.049	1.68	0.73–3.90	0.224
*Terai*	0.53	0.30–0.95	0.034	0.85	0.48–1.50	0.578	0.79	0.36–1.73	0.557
Disadvantaged/remote community	0.69	0.42–1.15	0.155	0.86	0.51–1.43	0.553	0.71	0.35–1.43	0.339
Caste/ethnicity: Brahmin/Chhetri	1.56	0.98–2.50	0.063	1.26	0.81–1.96	0.300	0.98	0.54–1.80	0.959
Household owns a radio	1.17	0.76–1.82	0.469	1.31	0.87–1.98	0.197	0.87	0.51–1.49	0.623
Household equity quintile (ref: lowest)									
Second lowest	1.65	0.86–3.13	0.129	1.49	0.81–2.75	0.199	1.41	0.49–4.02	0.526
Middle	1.70	0.91–3.14	0.093	1.40	0.74–2.63	0.303	1.11	0.39–3.11	0.846
Second highest/highest	1.65	0.91–2.99	0.100	1.22	0.68–2.19	0.501	2.34	0.84–6.50	0.103

## 4 DISCUSSION

This study estimates associations between different adult family members' exposure to multi‐sectoral nutrition interventions, controlling for socio‐economic and demographic characteristics, and household adoption of promoted maternal and child nutrition behaviours, among households in the 1000‐day period in Nepal. Two years after the start of the intervention, after roll‐out of the family approach, three‐fourths of mothers and over one‐third of male household heads were exposed to at least one of three types of interventions: interactions with a *Suaahara II* frontline worker in the past 6 months, participation in a community event such as a health mothers' group meeting or a food demonstration, or listening to the *Suaahara II* radio programme, known as *Bhanchhin Aama*. Positive associations were found between maternal exposure to *Suaahara II* and all three outcomes: minimum dietary diversity, minimum acceptable diet, and increased child feeding when the child is sick. Male head of household exposure to *Suaahara II* was associated with increased feeding to a sick child, but not the other two outcomes. Furthermore, in models limited to households where the mother was exposed to *Suaahara II*, the addition of male household exposure was positively associated with increased feeding to the child during illness.

The prevalence of maternal exposure to *Suaahara II* was much greater than that of male household heads, which is consistent with the fact that programmatic interventions during the first phase of *Suaahara* and beginnings of *Suaahara II* were focused on mothers while the family approach was being designed and formative research conducted. The initial interventions may not have been perceived by fathers and other male adults in the household as relevant and worth the effort to engage. Similarly, (predominantly female) frontline workers may not have approached males as often either for interpersonal communication or to encourage community event participation; despite training and the explicit transition to a family approach, this seems to have remained the case. Specific programme activities, such as the men only community groups shown effective in Bangladesh recently, may be necessary to generate engagement among male household heads (Phuong Hong Nguyen et al., 2018). Greater exposure by mothers may reflect variation in interest, time, and division of roles among family members. In Nepal, many of these MCHN outcomes have traditionally been considered the mother's responsibility, and encouraging other household members, specifically men, to engage in conversations with a frontline worker, to attend a food demonstration about complementary feeding or to listen to a radio programme about maternal and child nutrition may take more time. Previous research has shown that those without prior expertise and authority in these domains may not be viewed by others in the household as having a decision‐making role (Aubel, 2012). In this same dataset, for example, 9 out of 10 mothers reported to have all or nearly all decision‐making control for child feeding and child health; among male household heads, one‐third reported control for child feeding and almost two‐thirds for child health. Similarly, not even 10% of young children are fed by someone other than the mother and among those with problems breastfeeding or complementary feeding, not even 10% discussed this with their spouse. These findings highlight that overall in Nepal, unlike other documented contexts, mothers are the major decision‐maker for child nutrition and mothers do not default to discussions with other adults in the family when faced with related challenges (Aubel, 2012; Bootsri & Taneepanichskul, 2017).

Maternal exposure was positively associated with all IYCF outcomes, which likely reflects that the intervention had time to roll‐out and intensify in the communities. This is consistent with prior studies on the effectiveness of multi‐platform social and behaviour change interventions (Bhandari et al., 2004, 2005; Cunningham et al., 2017; Dewey & Adu‐Afarwuah, 2008; Nguyen et al., 2016; Suresh et al., 2019). Furthermore, the positive association between male household head exposure and an IYCF outcome is consistent with other studies (Abbass‐Dick, Brown, Jackson, Rempel, & Dennis, 2019; Bilal et al., 2016; Mahesh et al., 2018; Mitchell‐Box & Braun, 2013; Mukuria, Martin, Egondi, Bingham, & Thuita, 2016; Raeisi, Shariat, Nayeri, Raji, & Dalili, 2014). This finding in 2019, after *Suaahara II* refined its approach to include engaging men in community events and added additional family members into the drama series on *Bhanchhin Aama* is encouraging, but the lack of finding of association for the other key IYCF outcomes raises questions. Perhaps more time or engagement of men in more than one platform is needed or perhaps men are not major barriers to adoption of these ideal behaviours and resources would best be allocated elsewhere.

Another interesting finding is that for both mothers and male household heads, exposure to *Suaahara II* interventions was most strongly associated with appropriate sick child feeding above other IYCF outcomes. This relationship between programme exposure and this particular behaviour is consistent with other published studies, using different *Suaahara* datasets (Choufani et al., 2020; Cunningham et al., 2017). For male household heads, this was the only significant association. In all cases, it is also the largest coefficient, even showing up to a tripling of the odds in households where both the mother and male household head are exposed. It could be that this behaviour is more doable because it is a one off, shorter time frame behaviour and does not require changes be made every day or permanently. It could also be that appropriate sick child feeding is seen as more urgent, as it is a curative behaviour, whereas breastfeeding and complementary feeding practices are viewed as preventative for the invisible problem of malnutrition. Finally, culturally, daily childcare in Nepal is gendered and considered to be the responsibility of women, but men's involvement in caring for someone who is sick is culturally accepted. Therefore, as men learn the importance of this behaviour through exposure to *Suaahara* interventions, they are more easily able to adapt this behaviour than others.

This quantitative study contributes to the emerging evidence base on family approaches to interventions and their impacts. There are several limitations that should be kept in mind when interpreting the findings. First, this is a cross‐sectional dataset and therefore directionality cannot be confirmed. Directionality, however, would not be expected to vary by household member, and this comparison between household members is the primary purpose of this study. Second, *Suaahara II* is highly aligned and integrated with government platforms and programming, which presents challenges for measurement of exposure to *Suaahara II*. For example, many community activities are hosted through health mothers' groups which have existed before the intervention in some communities; *Suaahara II* has worked to revitalize this platform and strengthen its quality. To include health mothers' groups as part of the *Suaahara II* exposure may misclassify some unexposed as exposed, whereas to not include health mothers' groups as part of the definition may misclassify some exposed as unexposed. These measurement challenges, however, are reflected in all the models regardless of which individual's exposure is being assessed and therefore should not influence the comparison of maternal versus male household head exposure. Third, the definition of exposure was perhaps not precise enough and given low overall exposure, did not enable assessment of intensity, which is important for assessing these associations (Choufani et al., 2020). Another related drawback is that all interventions, given how many there are and how they vary across implementation areas, were not measured in the survey; some additional family approach interventions, for example, that were also rolled out by *Suaahara II*, such as the selection of male GESI champions in select communities and a Letter to the Father distributed during antenatal care visits, would also be important to include in these analyses.

Consensus has yet to emerge on the definition of a family approach, yet it is vital that the concept is clarified so that the global development community is clear regarding who should be engaged, at what point, and how (Martin et al., 2020). It is also important for donors, governments, and implementers to consider what proof of the family approach model is needed to justify additional resources required to reach additional family members. Additional research, in Nepal and other settings, is also needed to further understand how programmes can best engage using a family approach; how these interventions and exposure can more precisely be measured; and what outcomes or perhaps intermediary outcomes can be expected to benefit from a family approach. Remaining research questions include whether joint or separate counselling of various family members is most effective, what key messages are most appropriate for each family member and how to prevent unintended negative consequences. Further implementation research will be needed to document and assess how these interventions engage the whole family and learn lessons regarding what works. Rigorous evaluations are needed, including costing components, to further guide discussions among implementers regarding adoption of the family approach, particularly in large‐scale programmes that also aim to adopt sustainable, scalable intervention approaches. This study contributes to the emerging evidence base on the effectiveness of engaging family members, specifically men, in interventions aiming to address poor child nutrition practices. Improving child nutrition outcomes in Nepal and other low‐income countries is vital for further reductions in child undernutrition. Donors, governments and implementers' combined efforts to engage in implementation science studies could identify the most appropriate intervention designs, including target populations for improving IYCF practices and in turn reducing undernutrition.

## FUNDING INFORMATION

Suaahara II, funded by the United States Agency for International Development (USAID) Cooperative Agreement AID‐367‐A‐16‐00006, provided financial support for this survey and analysis. This study is made possible by the generous support of the American people through USAID. The contents are the responsibility of the authors and do not necessarily reflect the views of USAID or the United States Government.

## CONTRIBUTIONS

KC and DN designed the study. RA, KC, PG and DN participated in data cleaning and statistical analyses. KC and DN prepared the first draft of the manuscript. RA, KC, PG, DN and SS provided revisions and additional text to manuscript drafts. KC and DN prepared the final manuscript, and all authors read and approved the final version for submission.

## CONFLICTS OF INTEREST

The authors declare that they have no competing interests.

## Supporting information


**Table S1:** Infant and young child feeding outcomes among sampled households and minimum detectable effect size, 2019

## Data Availability

The data that support the findings of this study are available on request from the corresponding author. The data will be made publicly available via USAID's Development Data Library after the end of the Suaahara programme.
