# Supplementary material for: Associations between parents' exposure to a multisectoral programme and infant and young child feeding practices in Nepal
Source: Matern Child Nutr. 2021 Jul 9;17(Suppl 1):e13143. doi: 10.1111/mcn.13143 (PMC8269143; doi:10.1111/mcn.13143)
Supplement: Supplementary file 1 — Table S1: Infant and young child feeding outcomes among sampled households and minimum detectable effect size, 2019 [file MCN-17-e13143-s001.docx]

| **Supplementary Table 1: Infant and young child feeding outcomes among sampled households and minimum detectable effect size, 2019** | | | | | | |  | |  | |  | | |  | |  |  |
| --- | --- | --- | --- | --- | --- | --- | --- | --- | --- | --- | --- | --- | --- | --- | --- | --- | --- |
|  | **All: Maternal exposure** | | | | **Households with male household head (HHH): Male HHH exposure** | | | | | | | **Among households with exposed mothers, households with male household heads: Male HHH exposure** | | | | | |
|  | **N=1826** | | **MDES** | **N=941** | | | | **MDES** | | **N=676** | | | | | **MDES** | | |
|  | **Exposed** | **Unexposed** |  | **Exposed** | | **Unexposed** | |  | | **Exposed** | | | **Unexposed** | |  | | |
|  | **%** | **%** |  | **%** | | **%** | |  |  | **%** | | | **%** | |  |  |  |
| **Child health and nutrition practices** |  |  |  |  | |  | |  | |  | | |  | |  | | |
| Exclusive breastfeeding | 69.0% | 68.3% | 0.09 | 71.4% | | 72.0% | | 0.12 | | 72.4% | | | 68.8% | | 0.12 | | |
| Early initiation of breastfeeding: within an hour of birth | 75.8% | 71.0% | 0.05 | 75.7% | | 78.1% | | 0.05 | | 74.4% | | | 82.0% | | 0.06 | | |
| Minimum dietary diversity | 61.9% | 43.9% | 0.06 | 65.7% | | 52.9% | | 0.07 | | 69.8% | | | 59.8% | | 0.08 | | |
| Minimum acceptable diet | 51.1% | 35.1% | 0.06 | 52.2% | | 43.2% | | 0.08 | | 55.4% | | | 49.1% | | 0.09 | | |
| Sick child feeding: more during illness | 39.3% | 25.7% | 0.08 | 45.8% | | 29.4% | | 0.10 | | 49.4% | | | 29.8% | | 0.14 | | |
